# Scoping review of the recommendations and guidance for improving the quality of rare disease registries

**DOI:** 10.1186/s13023-024-03193-y

**Published:** 2024-05-06

**Authors:** JE Tarride, A. Okoh, K. Aryal, C. Prada, Deborah Milinkovic, A. Keepanasseril, A. Iorio

**Affiliations:** 1 Department of Health Research Methods, Evidence and Impact, Faculty of Health Sciences, McMaster University, Hamilton, Canada; 2 Centre for Health Economics and Policy Analysis (CHEPA), McMaster University, Hamilton, Canada; 3 Programs for the Assessment of Technologies in Health (PATH), The Research Institute of St. Joe’s Hamilton, St. Joseph’s Healthcare Hamilton, Hamilton, ON Canada

**Keywords:** Rare diseases, Registries, Quality standards, Guidance

## Abstract

**Background:**

Rare disease registries (RDRs) are valuable tools for improving clinical care and advancing research. However, they often vary qualitatively, structurally, and operationally in ways that can determine their potential utility as a source of evidence to support decision-making regarding the approval and funding of new treatments for rare diseases.

**Objectives:**

The goal of this research project was to review the literature on rare disease registries and identify best practices to improve the quality of RDRs.

**Methods:**

In this scoping review, we searched MEDLINE and EMBASE as well as the websites of regulatory bodies and health technology assessment agencies from 2010 to April 2023 for literature offering guidance or recommendations to ensure, improve, or maintain quality RDRs.

**Results:**

The search yielded 1,175 unique references, of which 64 met the inclusion criteria. The characteristics of RDRs deemed to be relevant to their quality align with three main domains and several sub-domains considered to be best practices for quality RDRs: (1) governance (registry purpose and description; governance structure; stakeholder engagement; sustainability; ethics/legal/privacy; data governance; documentation; and training and support); (2) data (standardized disease classification; common data elements; data dictionary; data collection; data quality and assurance; and data analysis and reporting); and (3) information technology (IT) infrastructure (physical and virtual infrastructure; and software infrastructure guided by FAIR principles (Findability; Accessibility; Interoperability; and Reusability).

**Conclusions:**

Although RDRs face numerous challenges due to their small and dispersed populations, RDRs can generate quality data to support healthcare decision-making through the use of standards and principles on strong governance, quality data practices, and IT infrastructure.

**Supplementary Information:**

The online version contains supplementary material available at 10.1186/s13023-024-03193-y.

## Introduction

Randomized clinical trials (RCTs) for many years have been the main source of clinical evidence for regulatory and reimbursement decisions of healthcare technologies. However, as regulators and health technology assessment (HTA) agencies move towards a life cycle approach [1, 2], there is an opportunity to broaden the evidence base and enhance decision-making through the integration of real-world evidence (RWE) into decision making. Based on real world data (RWD), RWE allows decision-makers to better understand how health technologies are being used, how they perform, and whether they are cost-effective in real-world healthcare settings. It is therefore not surprising that several frameworks have been developed over the past years to guide the use and reporting of RWD for decision making [3–9]. For common diseases, RWE is often provided by post-marketing phase IV clinical trials, administrative databases, or electronic medical records. In the case of rare diseases (RDs) characterized by small populations (e.g., fewer than one in 2,000 as per the Canadian or European definitions, or fewer than 200,000 in the US) [10], both traditional trials and common sources of RWD may be not providing sufficient evidence. For example, it may also not be feasible or ethical to conduct clinical trials for RDs [11–13]. Therefore, high-quality rare disease registries (RDRs) can play an important role in HTA, health policy, and clinical decision-making for RDs [14, 15]. RDRs can improve our knowledge of RD conditions, support clinical research, improve patient care, and inform overall healthcare planning. However, RDRs are often diverse in nature, supported by different data governance and funding models, and may lack standardized data collection methods [16]. As such, HTA agencies may be reluctant to use RDR data to inform funding decisions on treatments for rare diseases [17, 18].

To support acceptance of registry data by HTA bodies wishing to use registry data, the European Network for Health Technology Assessment (EUnetHTA) Joint Action 3 led the development of the “Registry Evaluation and Quality Standards Tool” (REQueST) [19] based on the Methodological Guidance on the Efficient and Rational Governance of Registries (PARENT) guidelines [20] and a series of HTA consultations [17, 20]. Although not specific to RDRs, the REQueST tool includes 23 criteria to support the assessment of whether registries meet the needs of the regulatory and HTA bodies including eight criteria describing the methodology used (type of registry; use for registry-based studies and previous publications; geographical and organizational setting; duration; size; inclusion/exclusion criteria; follow-up and confounders), 11 criteria that are essential standards for good practices and data quality (registry aims and methodology; governance; informed consent; data dictionary; minimal data set; standard definitions; terminology and specifications; data collection; quality assurance; data cleaning; missing data; financing; protection; and security and safeguards) and three criteria that deal with information that may be required when evaluating a registry for a particular purpose (e.g., interoperability and readiness for data linkage; data sources; and ethics). The REQueST tool was piloted with two established European registries and results indicated that both registries performed well, with more than 70% of the domains rated satisfactory and none of the domains failed. However, results indicated that more information was required in terms of governance structure (e.g., the role of industry), data quality checks, and interoperability [17].

The REQueST tool was also used by the Canadian Agency for Drugs and Technologies in Health (CADTH) to describe 25 RDRs based on publicly available information reported by the RDRs [21]. Within the study limitations (e.g., an assessment with the REQueST tool should be completed by registry data holders and not based on public information), the results indicated that most Canadian RDRs scored well for the 8 methodological criteria, although no RDRs provided public information on methods used to measure and control confounding. While information on the RDR purpose, governance, and informed consent was publicly available for almost all RDRs, there was considerable variation in the amount of publicly available information on the other REQueST criteria for the 25 Canadian RDRs, thus prompting a call for the establishment of Canadian standards for RDRs [21]. Therefore, to support decision making around the approval or funding of treatments for RDs in Canada and elsewhere, the objective of this study was to identify best practices to improve the quality of RDRs.

## Methods

A scoping review was conducted to meet the study objectives, as scoping review designs are particularly appropriate to answer broad research questions [22]. The scoping review included four steps: (1) developing the literature search strategy; (2) study selection; (3) data charting; and (4) summarizing and reporting the results.

### Search strategy

The search strategy was developed by a librarian from CADTH. The search strategy (Appendix 1) included several search terms (e.g., rare disease, registry, recommendations, guidance, standards). Databases searched were MEDLINE and EMBASE and the search was restricted to articles published in English from 2010 to April 2023. The year 2010 was chosen as the cut-off point because 2010 corresponds to the guidance on RDRs published by the European Rare Disease Task Force initially published in 2009 and updated in 2011 [23]. Grey literature was searched from websites of regulatory bodies (e.g., European Medicines Agency, Food and Drug Administration, Health Canada) and HTA authorities (e.g., National Institute for Health and Care Excellence, CADTH).

### Study selection

Screening for articles that met the inclusion was conducted using Rayyan [24]. Titles and abstracts were screened against the inclusion and exclusion criteria (Level I screening). Full texts of the publications that passed the Level I screening were retrieved before being screened for final inclusion and exclusion (Level II screening). The literature was screened by two pairs of independent reviewers (KA & CP; AK & AO) at each stage of the Level I and Level II screenings. Conflicts within each pair of reviewers were resolved through discussion. When consensus could not be reached an additional reviewer was consulted (JET). The same process was used for screening the grey literature.

### Inclusion and exclusion criteria

Literature was included if it was reporting on standards, processes, guidance, or recommendations for improving the quality of RDRs. Exclusion criteria included: (1) non-English literature; (2) conference proceedings and letters; and (3) papers presenting clinical data based on an existing RDR without reporting on standards, guidance, or considerations relevant to RDR quality. The references cited in the included papers were also scanned to identify any relevant literature, including non-RDR guidance cited in the RDR literature.

### Data charting

Based on the preliminary scoping of the literature, the following data were selected for abstraction: publication details and specific guidance related to RDRs’ governance; patient engagement and consent; diversity and equity issues; funding model and sustainability; ethical/legal/regulatory requirements; data quality and management; data elements; standardization; data linkage; data validity and audit; IT infrastructure; and barriers and facilitators for improving the quality of RDRs. Data was abstracted into a Microsoft Excel spreadsheet.

### Data summary and synthesis

Once the data were abstracted, summaries were created by the team and the information was synthesized in terms of best practices for improving the quality of RDRs.

## Results

### Results of the search strategy

**Fig. 1:**
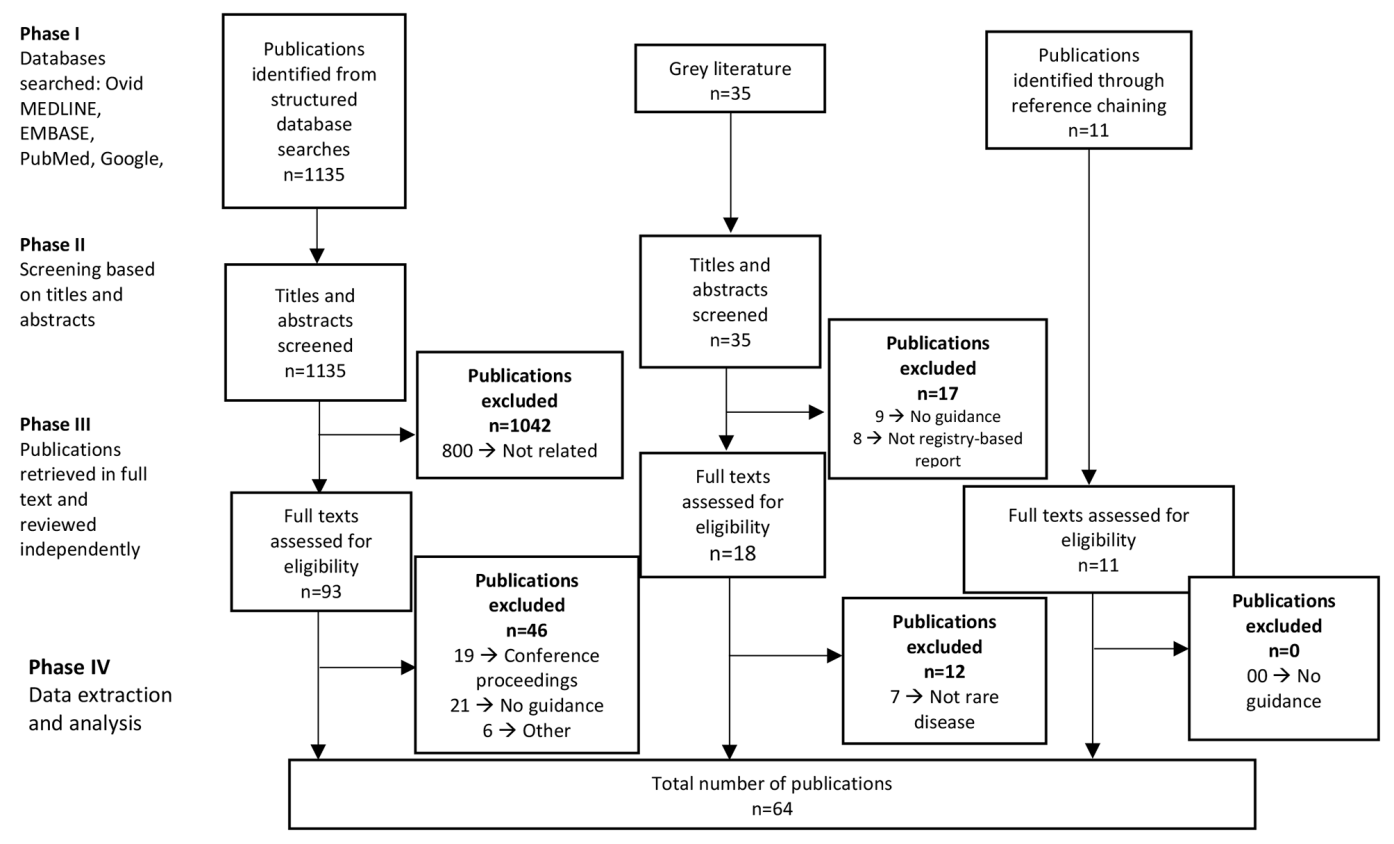
PRISMA diagram

Out of 1,135 unique citations identified by the search, 93 were assessed for eligibility based on a full-text review, and 47 studies were included for data abstraction. For the grey literature, 35 documents were identified, 18 were assessed for eligibility based on full-text review and 6 documents were included for data abstraction. In addition, 11 documents were identified by reviewing the references cited in the included papers, for a total of 64 documents included in our scoping review. Figure 1 presents the PRISMA diagram summarizing the screening process and key reasons for exclusion. Appendix 2 presents the list of the 64 documents identified through the literature review and used to develop the framework.

### Conceptual framework

Upon review of the evidence and authors’ discussion, the literature was synthesized according to three key quality domains (governance, data, and information technology) and several sub-domains: eight for governance (registry purpose and description; governance structure; stakeholder engagement; sustainability; ethics/legal/privacy; data governance; documentation and training and support), six for data (standardized disease classification; common data elements; data dictionary; data collection; data quality and assurance; and data analysis and reporting), and two for IT infrastructure (physical/virtual infrastructure; and software infrastructure).

### Domain 1: RDR governance

Governance was the most discussed domain (48 of 64 sources), which was not surprising given that governance is foundational for quality and trust. Governance refers to the formalized structure that guides the RDR leadership and high-level decision-making required to achieve RDR’s objectives and long-term operational sustainability [13, 16, 25]. The following describes the guidance reported in the literature for each of the 8 governance sub-domains, while Table 1 summarizes the key guidance.

#### Sub-domain 1 — registry purpose and description

The critical first step in any registry description is to state its purpose and objectives since they establish the framework for all activities that follow (e.g., data collection, inclusion, and exclusion criteria). A comprehensive description of the registry, available through the registry website or publications, allows other stakeholders, including potential researchers or regulatory or HTA users, to understand and appraise the registry’s quality and potential usefulness. In addition to the RDR purpose and objectives, common attributes reported in the literature to describe a registry include registry design, timeframe, population characteristics, settings, geographical coverage area, type of data captured and data sources, data quality procedures, data access policies, ethics approvals and dissemination activities [17, 20, 23, 25–42].

#### Sub-domain 2 — governance structure

The governance structure reflects the nature and extent of registry operations [13]. As for many organizations, the adoption of an organigram (a visual representation of the registry governance structure) helps clarify roles and responsibilities, reporting and decision-making flow, and how the different roles interconnect [29, 43]. Examples of key roles and expertise reported in the RDR literature include: registry lead(s); project manager(s) and management team with financial and leadership experience; information technology experts; data entry personal; and team members with specific expertise (e.g., ethics, legal, statistics, population-based research) [25, 31, 37, 38, 42, 44]. A central contact point for stakeholders is advisable [18, 38, 45].

Depending on the size and scope of the registry, a governing body, spanning from an independent Board of Directors to a Steering Committee comprised of various internal and external experts, has been recommended [35, 36]. The role of the governing body is to direct daily operations and ensure compliance with applicable laws and regulations, directly or through small targeted work groups [42, 45, 46]. In addition, independent advisory boards can provide technical guidance and scientific independence [13, 23, 47]. Patient representation on governing bodies or committees facilitates patient centeredness and engagement in the decision-making process [17, 20, 25]. As for most public and private organizations, board and committee members should declare conflicts of interest to enhance transparency [17, 35, 48].

#### Sub-domain 3 — stakeholder engagement

Multi-stakeholder engagement (e.g., clinicians, patients and their families, patients organizations, provider organizations, regulators, payers, drug companies) is suggested to facilitate long-term sustainability of RDRs [23, 25, 37, 38, 41, 46, 49–53]. The integration of a broad group of stakeholders can also facilitate quality improvements [23, 30, 50, 54]. Patient advocacy groups, for example, can enhance the accuracy and completeness of patient data [49]. However, the literature points out that decision-making may be challenging with a large number of stakeholders [25].

#### Sub-domain 4 — sustainability

Durable and long-term sustainability is dependent upon funding and is key to ensuring RDR quality [25, 36]. Compared to non-RD registries that have large populations to draw upon, RDRs are constrained by small and dispersed patient populations and limited funding opportunities. These constraints can inhibit data accuracy, patient follow-up, standardization, and result in knowledge gaps [26, 29, 30, 52, 54]. Multiple funding sources (e.g., public or private organizations, public-private partnerships, non-profit foundations, patient groups, professional societies) may contribute to the long-term sustainability of RDRs [25, 35, 44, 49], but for transparency, it is important that all funding sources are publicly disclosed [17, 18, 45, 50]. Sustainability also necessitates a long-term comprehensive financial plan including future-oriented exit strategies and succession planning [39, 47, 50]. A registry’s utility, effectiveness, efficiency, and agility are also important to ensure its long-term sustainability [27, 55].

#### Sub-domain 5 – ethics, legal, privacy

RDRs must comply with ethical, legal, and privacy regulations [25, 32, 33, 35, 36, 39, 42] for the collection, storage, and use and re-use of patient health data for RDR activities [23, 27–30, 34, 38, 45] or for regulatory purposes [12, 45]. The collection of informed consent necessitates that participants understand the risks and benefits that might accrue to them specifically, who might have access to their data, how their data will be used and re-used including potential linkages to other registries or future research activities, and a participant’s right to withdraw their consent at any time [17, 20, 45, 50, 56]. Since the withdrawal of consent impacts both the current data holdings and past, present, and future research analyses, precise language in the consent about the withdrawal consent (e.g. what happens to the data of an individual withdrawing consent and how it impacts the analyses) will help mitigate potential misunderstanding and future conflicts [13]. Approaches used to encourage participation, if any (e.g., incentives) should be documented [26]. For international registries or RDRs intending to link to international registries, multiple statutes may apply (e.g., EU General Data Protection Regulation (GDPR), Canada’s Personal Information Protection and Electronic Documents Act (PIPEDA), various in the U.S.) [57, 58]. For this reason, it is often recommended that RDRs strive to comply with international, national, and local ethical, legal, and privacy regulations as appropriate [23, 25, 36, 49, 50]. 

#### Sub-domain 6 - data governance

Data ownership and data custodianship are at the forefront of data governance [16, 35, 36, 38, 48, 59]. Data ownership refers to the possession, responsibility, and control of data including the right to assign access privileges to others [60]. Patient participants may grant the registry authorization to access and use their data for research [16, 29, 38]. However, more than one entity (e.g., patients, clinicians, hospitals, funders) could have a claim to the aggregate data in the registry [16, 18, 38, 46]; therefore, data ownership must be clearly defined. Data custodianship is the responsibility of the registry organization, which includes monitoring and managing registry use, data access policies, and data sharing agreements [18, 45, 46]. A protocol for third-party data requests, such as the administration of these requests through a data access committee, will ensure that requests are appropriately assessed and responded to in a timely manner [16]. Full disclosure of the registry’s fee structure (e.g., fees for ad-hoc requests versus subscription fee models) will mitigate potential miscommunication or misinterpretation of data access requirements [26].

#### Sub-domain 7 — documentation

Documentation is essential to maintaining a quality registry because it facilitates shared understanding and transparency around the registry activities. A Standard Operating Procedures (SOPs) manual that is updated regularly provides step-by-step guidance on the registry’s routine activities including performance targets [25, 30, 35, 38, 61]. Regular provision of activity reports (e.g., annual reports) and a repository of registry-based publications increase the transparency of the RDR processes and activities [17, 25, 48, 62]. Similarly essential is the documentation of ethical and regulatory approvals for registry-based studies [12, 32, 33, 47] and the adoption of standardized templates and forms (e.g., informed consent) that reflect the registry’s objective and use standardized language [33, 40, 61]. The adoption of an Investigator and User Declaration Form or similar document will affirm compliance with regulatory and operational processes [32]. The literature also recommends publishing study protocols and registering registry-based studies in a public register [18, 45].

#### Sub-domain 8 – training and support

Training is essential for registry staff, data providers, and new users to ensure consistency and quality [25, 38, 45, 63]. A training manual, “how-to videos”, and a comprehensive training plan that are updated regularly facilitate consistent training protocols [25, 37, 54]. A registry might also benefit from designated data entry personnel who can systematically monitor and evaluate data quality [38]. A Support Team or Help Desk is also beneficial to the operations of the registry [59].

### Domain – data

Data was the second most discussed domain (45 of 64 sources). Data refers to the structures, policies, and processes required to ensure a RDR can maintain a high-quality database [13]. A high-quality database is characterized by completeness, accuracy, usefulness, and representativeness [13, 25, 64], which is paramount for meeting the needs of decision-makers.

Table 2 summarizes the guidance reported in the literature, which is described in more detail below in terms of 6 sub-domains (standardized disease classification; common data elements; data dictionary; data collection; data quality and assurance; data analysis and reporting).

#### Sub-domain 1 – standardized disease classifications

Standardized disease classifications such as the Orphanet Rare Disease Ontology (ORDO), Human Phenotype Ontology (HPO), ORPHA-codes or the International Classification of Disease ICD-9, ICD-10, or ICD-11 or some combination [31, 32, 50, 65, 66] have been proposed for data collection to ensure future interoperability and registry linkages. Being able to link to other registries facilitates knowledge creation, decision making, and improvements in clinical care that may not otherwise be possible for small RD patient populations [33, 36, 49, 51, 67]. When RDRs transfer or merge their data to or with other entities, the documentation of the process used to validate the data transfer ensures quality and consistency [68]. Although linking to international RDRs expands population reach, it poses some additional challenges (e.g., the regulatory environment) that need to be identified early in the registry design process [44]. The use of international standards and ontology codes apply in this context.

#### Sub-domain 2 – common data elements

Registries have to consider the informational needs of the registry against the needs of their other stakeholders and the available resources [25]. A minimum set of common data elements collected across the RDR sites (e.g., administrative data, socio-demographics, diagnosis, disease history, treatments, clinical and safety outcomes) that could be expanded upon to meet the specific needs of the registry is usually identified [37, 41, 50, 53, 56, 69]. Ideally, these common data elements would be harmonized across all registries that represent the same rare disease when applicable [12]. However, the main challenge around common data elements is reaching a consensus regarding the choice, organization, and definition of the various elements [25, 70, 71]. Beyond simply determining the composition of the common data elements, other challenges include data coding standards (e.g., integer, float, string, date, derived data, and file names) [13, 72, 73], standardized data constructs, vocabulary and terminology [28, 33, 37, 65, 71, 74], defined variable interpretation to avoid inconsistency (e.g., sex – genotypic sex or declared sex) [18, 75] and ontology harmonization to facilitate convergence from different terms or languages [56, 65, 76, 77]. The latter necessitates consistent agreed-upon disease classification standards [23, 50, 77].

#### Sub-domain 3 – data dictionary

A detailed data dictionary is an essential tool for quality data collection [17, 20, 25]. A data dictionary provides clear instructions for data entry and analysis by defining all data elements and their purpose as well as the coding values including permissible values, representation class, data type, and format [17, 20]. Complete alignment between the variables described in the data dictionary and those captured by the registry’s interface is expected [55].

#### Sub-domain 4 – data collection

Procedures for documenting the entire data collection process including adverse event monitoring, baseline and follow-up data, causality assessment, and reporting timelines are recommended to improve data accuracy [18, 51]. Standardized data collection forms (e.g., Clinical Data Interchange Standards Consortium Operational Data Model, Patient Records, and Outcome Management Information System) can facilitate the data collection process [35]. Training in data collection procedures is key to reducing information bias and data misclassification and to achieve consistency amongst users and high quality data collection [25, 35]. Sustained investment in data collection and management is also critical as prospective data collection across the patient’s lifespan can be expensive and onerous [78]. The capacity for a registry to embed clinical studies into its own database can also help sustain the registry and reduce costs associated with duplicated data collection efforts when conducting additional studies [31, 78].

Data collection tools such as computers, automation, smartphones, smartwatches, tablets, and medical devices (e.g., glucose monitors) can be valuable sources of electronic health data as well as increase registry participation, particularly from disparate geographic locations, which in turn can result in increased knowledge, improved patient outcomes, stronger patient advocacy, and enhanced equity through healthcare access [13, 37, 39, 40, 75, 79]. However, internet-based data collection may impact equity for data providers with limited access to the internet. Relationship building with physicians and patient groups who serve often excluded groups can facilitate greater equity and inclusion through referrals and knowledge translation efforts that promote and encourage registry participation [32, 40].

#### Sub-domain 5 – data quality and assurance

Data quality reflects various data attributes or dimensions that can be used to measure the calibre of the data [25] such as completeness (the extent that the stored data represents the potential data), uniqueness (no repeated or redundant data), timeliness (data is up to date at the time of release), validity (data conforms to the appropriate syntax [e.g., format, type, range]), accuracy (the data correctly reflects the object or event being described), consistency (there are no discrepancies when the data is compared across different databases or against its definition) [35, 64], and usefulness (the extent to which the outputs provide value) [25].

Data quality and assurance plans which include data validation (e.g., medical, clinical, and record audit) [61] and a review of RDR-generated studies ensure compliance with RDR-based studies’ protocols and ethical and regulatory requirements [12, 25, 32, 69]. Data quality and assurance processes necessitate routine data quality checks and data cleaning to ensure the enrolment of eligible patients, data completeness, validity, and coherence while mitigating record duplication and errors [26, 31, 35, 45, 55]. Data audits can be performed by internal registry staff or an external service provider, or some combination [37]. Regular feedback to data providers about these data quality activities and findings encourages prompt remedial action and learning, thus improving the quality of the RDR data [25, 45, 50].

#### Sub-domain 6 – data analysis and reporting

In addition to study protocols, RDR-based studies benefit from the development of statistical analysis plans (SAPs), whether for internal registry objectives or external research with third-party partners [12, 13, 25, 45]. A SAP facilitates the production of trustworthy results that can be more easily interpreted and accepted by various stakeholders (e.g., registry participants, patient groups, researchers, decision-makers, or the general public) [25]. SAPs should provide a list of variables and confounders captured in the data and details on the statistical methods used to answer the study question(s) and to deal with missing or censored data [25, 45]. Adoption of guidelines such as the Strengthening the Reporting of Observational Studies in Epidemiology (STROBE) Statement, or the Patient-Centered Outcomes Research Institute (PCORI) Methodology Report improves transparency and accuracy when reporting RDR findings [13]. Details about dissemination activities such as study reports and communication strategies are usually included in the study protocols [25, 44, 45].

### Domain – information technology infrastructure

Information technology (IT) infrastructure was discussed in 29 of the 64 documents in terms of physical and virtual infrastructure, and software infrastructure. IT infrastructure refers to the critical infrastructure that is required to collect, share, link, and use patient and clinical data [16, 32, 37], and importantly, securely store, transmit, and manage this private data [32, 33, 63]. Table 3 summarizes the literature which is described below in more detail below.

#### Sub-domain 1 – physical and virtual infrastructure

High quality RDRs are characterized by procedures and processes that ensure the digitally stored private data is secure [29, 32, 35, 62, 80] by housing the data on dedicated servers with intrusion detection systems [35]. Critical decisions include where the server(s) are held (e.g., centralized database versus distributed); how these location(s) are secured, and how and who has access to the RDR data. Registries can safeguard their systems through several processes (e.g., analysis of threats and countermeasures [63]) and tools (e.g. data software and access policies [63, 81, 82]). An independent external security or threat risk assessment is recommended to document compliance with security and privacy standards [83].

#### Sub-domain 2 – software infrastructure

The adoption of FAIR principles at the data source can facilitate data connections and exchanges across multiple RDRs and bolster data quality that supports both clinical research and patient care [31, 56, 68, 73, 75, 81]. FAIR data principles stand for Findability (easy to find for both humans and computers), Accessibility (easily retrievable and accessible by users), Interoperability (easily integrates with other data), and Reusability (well-described so it can be replicated or applied in another setting). Since FAIR principles enable the extensive and efficient use of registry data while mitigating duplication, recollection, and errors [25, 56], it is recommended that the registry data infrastructure complies with FAIR principles [18, 25, 26, 37, 38, 59, 75, 83, 84].

The technology choices, software architecture design and software development practices have a dramatic impact on software sustainability, legacy software support, ease of software modification, enhancements and interoperability [81]. With this in mind, software solutions can either be “out-of-the-box” commercial software or “home-built”, custom-designed in-house software, the latter often being more powerful but more resource and time-intensive [25]. Either way, drop-down menus, pop-up explanatory notes, and tab-to-jump options will aid in rapid and user-friendly data entry [32, 72]. A user-friendly web interface with the capacity to upload and download data can also facilitate data sharing [25, 38]. Since registry data is often collected from several sources, machine-readable files could facilitate the interoperability of pseudonymized data subsets, reduce duplication, and make the data more findable [26, 63, 67, 75]. However, data heterogeneity can prove a barrier to automation [79].

Regardless of the methods used to collect, store, and manage data sets, data encryption and firewalling of servers are standard [59, 63]. The encryption of data while in transit is an added layer of data security [27, 32, 40]. As it might become necessary to delete data occasionally (e.g., participants who revoke their consent), standardized procedures for data deletion help maintain database integrity and mitigate errors [13, 38, 41]. Enhanced technological literacy and adoption of technology tools (e.g., electronic health records, automated data capture, internet and mobile devices) [44, 54] and the integration of a broader group of stakeholders [23, 30, 54] can also facilitate quality improvements.

## Discussion

Because RDRs can be designed around different purposes (e.g., patient advocacy, enhanced clinical practice, epidemiological and research goals) [25, 41, 46], RDRs often vary in quality, and are structurally and operationally diverse [38]. As a result, their fitness for purpose as a source of data to support decision making around the approval or funding of treatments for rare diseases must be assessed on a case-by-case basis [17, 18]. Fortunately, the RDR literature offers a range of quality standards that define the essential characteristics leading to the development and maintenance of a quality RDR. The guidance from the 64 sources captured by the scoping review is synthesized within three dominant domains: (1) governance, which represents the many operational features of governance such as the governance structure, stakeholder engagement, sustainability, ethic and regulatory oversight, and training; (2) data, which represents standardized ontology and common data elements and standardized processes for data entry, verification, and auditing and reporting; and (3) information technology, which represents physical and software infrastructure and security, guided by FAIR principles.

While many guidelines focused on certain dimensions of RDRs’ quality (e.g., governance, core data elements), only three papers provided overall guidance on an extensive set of elements required to set up and maintain high-quality RDRs. Among those, in 2018, Kodra et al. 2018 [25] reported on the set of 17 recommendations to improve the quality of RDRs developed by a select group of experts convened by the Italian National Center of Rare Diseases in collaboration with other European countries [25]. These recommendations touched on 11 topics (registry definition; registry classification; governance; data sources; data elements; case report form; IT infrastructure complying with FAIR principles; quality information; documentation; training; and data quality audit) [25, 61]. Building on these recommendations and expert meetings, in 2020, Ali et al. [38] surveyed the RDR community to determine the consensus level regarding 17 criteria that could be considered essential when assessing the quality of a RDR in terms of registry governance (9 items), data quality (5 items), and IT infrastructure (6 items). The responses of 35 respondents representing 40 RDRs across the United States, Canada, the United Kingdom, and Europe indicated a high level of consensus among the RDRs with more than 90% of respondents agreeing with most of the 17 criteria. Of note, 30% of respondents did not feel that patient involvement in the registry governance was necessary, conceding that although patient involvement in RDR governance may be best practice, there may be a limited role for patients in some scenarios such as physician-driven registries. The 2021 European Medicine Agency (EMA) guidance [45] integrated guidelines from PARENT Joint Action Methodological Guidance [20], the EUnetHTA’s Registry Evaluation and Quality Standards Tool (REQueST) [19], the US Agency for Healthcare Research and Quality (AHRQ)’s Users’ Guide on registries [13], and the European Reference Network Patient Registry Platform [85]. The EMA guidance provides information regarding two main domains: Administrative Information (subdomains include governance, consent and data protection) and Methods (subdomains include objectives, data providers, population, data elements, infrastructure, quality requirements). Our review broadly aligns with all three of these sources in terms of content, but it is more closely aligned with Ali et al. 2021 in terms of organization and number of domains (registry governance, data quality, IT infrastructure). However, compared to these guidances based on consensus panels or surveys, the evidence leading to our framework was based on a scoping review of the literature and we synthesized a broader set of quality indicators deemed essential by the literature. In December 2023, the FDA released its finalized real-world data guidance regarding a registry’s fitness to support regulatory decision making, which is consistent with our framework (e.g., governance, data, information technology infrastructure) [86]. However, this guidance was published after the completion of the scoping review and as such was not included in this review.

Despite the literature on quality standards for RDRs, a review of 37 publications reporting on RDRs between 2001 and 2021 found that while most of these publications reported on collecting informed consent (81%) and provided information on data access, data sharing or data protection strategies (75%), fewer publications reported on quality management (51%) or maintenance (46%). Furthermore, fewer RDRs reported using core data elements (22%) or ontological coding systems (24%), which is key for interoperability and for linking registries [21]. It is however possible that RDRs had such policies in place but did not report on them. Initiatives such as those undertaken in Europe to develop guidance to improve the quality, reporting, and assessment of patient registries from both regulatory and HTA perspectives facilitate the integration of registry data into decision-making processes. For example, the REQueST tool has been used in Europe by HTA agencies to evaluate the quality of registries being used as a source of data to support decision-making [17]. However, the REQueST assessment of 25 Canadian RDRs based on publicly available information on these RDRs highlighted the importance of developing standards for Canadian RDRs [21]. In this context, the results of this scoping review could be used to help develop a Canadian consensus on the core standards defining high-quality RDRs from regulatory and HTA perspectives. Compliance with RWE guidance [87, 88] and acceptance of evidence from other jurisdictions are other important considerations when using RDR data for decision-making, especially in countries with relatively small populations such as Canada.

While adhering to existing and future RDR and HTA guidance will certainly improve the quality of RDRs and their use in decision-making, it should be recognized that this may require significant investment in terms of human and financial resources which may not be easily available to all RDRs. However, at least for Canada, the recent announcement in March 2023 by Health Canada to invest $1.5 billion over three years in support of a National Strategy for Drugs for Rare Diseases [89] represents a unique opportunity to develop a national infrastructure of sustainable, standardized, and quality RDRs while aligning with the pan Canadian Health Data Strategy [90].

As a next step, for RDRs interested in the harmonization of their data collection with other registries, the European Platform on Rare Disease Registration (EU RD Platform) [91]serves as an example of how this might be achieved. With over 600 diverse and fragmented rare disease registries in Europe, the European Commission’s Joint Research Centre in collaboration with stakeholders took on the tremendous task of establishing standards for integration, training, and interoperability of RDR data across Europe. A core element of the EU RD Platform is the European Rare Disease Registry Infrastructure (ERDRI) [92], which consists of a directory of registries, a data repository, a pseudonymization tool and importantly the EU RD Platform comprising of a set of 16 common data elements [93] that capture the characteristics of rare disease patients such as demographic, and clinical, diagnostic, and genetic information [43, 56, 76, 91, 94].

## Limitations

Before interpreting the results of this scoping review, several limitations should be considered. First, due to the unique characteristics of RDRs, we limited our scoping review to RDRs, and we did not search the literature to improve the quality of non-RD registries. However, we identified several non-RDR guidances [12, 13, 20, 45, 64, 68] when checking the references of the RDR papers included in the scoping review. Second, although we took a systematic approach when selecting the papers to be included in our scoping review, it is always possible that we missed one or several studies, even though all included publications’ references were checked to identify relevant studies not included in our final list of documents. Third, while we summarized the guidance under three domains and 16 sub-domains, we did not develop recommendations. This is left for future research. Despite these limitations, the results of this scoping review of 64 documents published between 2010 and April 2023 add to the body of the literature offering suggestions to improve the quality of RDRs. The results of this scoping review provide the foundation to develop quality standards for RDRs in Canada or other countries lacking guidelines for quality RDRs. For example, these results could be used by a Delphi panel to develop standards and processes to enhance the quality of data in RDR registries.

This review has also identified a few areas which merit further consideration. First, from a Canadian standpoint, future work is needed to develop a database of Canadian RDRs along with information on their key characteristics (e.g., purpose, population, funding) and information regarding their governance, data and IT infrastructure. Second, although the literature agrees on the importance of being able to link with international registries, it is also important to be able to link RDRs with health administrative databases to provide HTA agencies and decision makers with information on short and long-term outcomes, healthcare resource utilization, and expenditures associated with RDs. Similarly, issues of equity and diversity were discussed by only a few papers in the context of data collection methods to encourage patient participation [27, 40, 80], and relationships with physicians and patient groups working with disadvantaged groups [32, 40]. A broader RDR equity lens could be achieved by using equity tools such as the PROGRESS (Place of resident, Race, Occupation, Gender, Religion, Education, Socioeconomic status, Social capital) framework [95], which would facilitate a greater understanding of how equity-deserving populations are affected by RDs or represented in RDR registries. Finally, the integration of patients’ experiences and insights when designing and interpreting results is an important avenue of research to enhance the quality and acceptance of RDR studies by generating patient-centered RWE [96].

## Conclusion

Although RDRs face numerous challenges due to their small and dispersed populations, RDRs can generate quality data to support healthcare decision-making through the use of standards and principles on strong governance, quality data practices, and IT infrastructure.

**Table 1:** Literature guidance on RDR governance

Sub-domain
Registry purpose and description
• Registry purpose/objectives and description (e.g., design, timeframe, population characteristics [e.g., inclusion/exclusion criteria, sample size, representativeness], settings, geographical coverage area, health interventions considered in the registry, type of data captured and data sources, data quality procedures, data access policies, ethics approval, dissemination activities) [17, 20, 23, 25–42].
Governance structure
• The governance structure reflects the nature and extent of registry operation [13].
• Organigram outlining the roles and responsibilities within the registry [18, 23, 25, 29, 31, 37, 38, 42–45].
• Board of Directors or Steering Committee performing an oversight function [35, 36, 42, 45, 46, 48].
• Independent advisory board providing technical guidance and scientific independence (where appropriate) [13, 23, 47].
• Inclusion of patients in the decision-making process [17, 20, 25].
Stakeholder engagement
• Broad range of stakeholders across health facilities, regulators, patients, industry partners, etc [23, 25, 30, 37, 38, 41, 46, 49–54].
Sustainability
• Access to multiple funding sources to facilitate long-term sustainability [17, 18, 25, 35, 44, 45, 49].
• Long-term comprehensive financial plan [25–27, 29, 30, 36, 39, 47, 50, 52, 54, 55].
Ethics, Legal, Privacy
• Compliance with the relevant (international, national, or local) ethical, legal, and privacy regulations [23, 25, 32, 33, 35, 36, 39, 42, 49, 50].
• Informed consent for data collection, storage, use and reuse, data linkages, and right to withdrawal of consent [17, 20, 23, 27–30, 34, 38, 45, 50, 56].
• Approaches used to encourage participation, if any, e.g., incentives [26].
Data governance
• Clear definition and documentation of data ownership rights [18, 29, 35, 36, 38, 45, 46, 48, 59].
• Adoption of a managed access approach for third-party data requests [16, 26].
Documentation
• Standard Operating Procedures (SOPs) manual [25, 30, 35, 38, 61].
• Regular reports on its activities and repository of registry-based publications [17, 25, 48, 62].
• Standardized forms (e.g., informed consent, Investigator Declaration Forms) [33, 40].
• Ethical and other regulatory approvals for registry-based studies [12, 32, 33, 47].
Training and support
• Training resources for registry staff, data providers, and new users [25, 38, 45, 63].
• Support team or help desk [59].

**Table 2:** Literature guidance on RDR data

Sub-domain
Disease classification standardization
• Adoption of standardized disease classifications to facilitate interoperability and registry linkages [31, 32, 50, 65–67, 77].
Common data elements
• Minimum or core group of data elements which can be expanded to meet the informational needs or objectives of the registry [37, 41, 50, 53, 56, 69, 76].
• Process describing how to define and organize the core data elements [13, 25, 70, 71, 74, 75, 77].
Data dictionary
• Data dictionary provides clear instructions for data entry [17, 20, 25].
• Complete alignment between the data dictionary and the data being collected [55].
Data collection
• Data collection procedure and standardized data collection forms [18, 35, 51].
• Adequate staff training in data collection procedures to minimize measurement errors [35].
• Data collection methods are flexible enough to promote equitable access to different demographics [13, 32, 37, 39, 40].
• Adequate funding to facilitate data collection across a patient’s lifespan [78].
Data quality and assurance
• Data quality and assurance plan [12, 25, 32, 61, 64, 69].
• Performing routine data quality checks and data cleaning [26, 31, 35, 45, 55].
• Provision of regular feedback to data providers on data quality activities and registry-based research findings [25, 45, 50].
Data analysis and reporting
• Study protocols inclusive of statistical analysis plans for registry-based studies [12, 13, 25, 45].
• Adoption of reporting guidelines (e.g., STROBE, PCORI) to improve reporting transparency and accuracy [13].

**Table 3:** Literature guidance on information technology infrastructure

Sub-domain
Physical and virtual infrastructure
• Secure storage of data [29, 32, 35, 62, 80].
• Adoption of independent external security or regular threat risk assessments [82, 83].
Software infrastructure
• Compliance with FAIR principles at the data source to improve data quality and enhance data connections and exchanges across multiple RDRs [18, 25, 26, 31, 37, 38, 56, 59, 68, 73, 75, 81, 83, 84].
• User-friendly web design and data entry portal, when applicable [32, 72] ideally allowing upload and download of data [25, 38].
• Adoption of encryption and firewalling of registry data [27, 32, 40, 59, 63].
• Provision and procedures to scale up the use of technological tools to enhance registry quality [44, 54, 67, 79].
• Promotion of technological literacy among its stakeholders to facilitate quality improvement [23, 30, 54].

### Electronic supplementary material

Below is the link to the electronic supplementary material.

Supplementary Material 1

Supplementary Material 2

Supplementary Material 3

Supplementary Material 4

## Data Availability

The authors confirm that a complete list of the sources used for data analyzed during this study is available in Appendix 2 of this published article. Example: 10.3390/ijerph15081644.
