# Supplementary material for: Scoping review of the recommendations and guidance for improving the quality of rare disease registries
Source: Orphanet J Rare Dis. 2024 May 6;19:187. doi: 10.1186/s13023-024-03193-y (PMC11075280; doi:10.1186/s13023-024-03193-y)
Supplement: Supplementary file 1 — Supplementary Material 1 [file 13023_2024_3193_MOESM1_ESM.docx]

**SCOPING REVIEW OF BEST PRACTICES FOR IMPROVING THE QUALITY OF RARE DISEASE REGISTRIES**

List of Abbreviations:

RDRs – rare disease registries

RDs – rare diseases

RTCs – randomized control trials

HTA – health technology assessment

RWE – real-world evidence

RWD – real world data

EUnetHTA – European Network for Heath Technology Assessment

REQueST – Registry Evaluation and Quality Standards Tool

CADTH – Canadian Agency for Drugs and Technologies in Health

SOPs – Standard Operating Procedures

ORDO – Orphanet Rare Disease Ontology

HPO – Human Phenotype Ontology

ICD – International Classification of Disease

SAPs – statistical analysis plans

STROBE – Strengthening the Reporting of Observational Studies in Epidemiology

PCORI – Patient-Centered Outcomes Research Institute

IT – Information technology

FAIR – Findable, Accessible, Interoperability, Reusability
