# Supplementary material for: Scoping review of the recommendations and guidance for improving the quality of rare disease registries
Source: Orphanet J Rare Dis. 2024 May 6;19:187. doi: 10.1186/s13023-024-03193-y (PMC11075280; doi:10.1186/s13023-024-03193-y)
Supplement: Supplementary file 4 — Supplementary Material 4 [file 13023_2024_3193_MOESM4_ESM.docx]

**Appendix 1: search strategy**

**Limits: 2010-** , English and French

**Databases:** Medline and Embase

**Set#1 – Registry Standards**

((registry or registries) ADJ4 (establish* or development or standards* or model* or developing or design or framework* or build* or implement*)).ti.

(EPIRARE or Open-Source Registry System for Rare Diseases or (OSSE ADJ2 framework)).ti,ab.

**Set#2 – Registries (general)**

Registries/ OR exp patient registry/

(registry or registries).ti,ab,kf.

**Set#3 – Standards**

*Quality Control/ or Data Accuracy/ or Common Data Elements/ or *Metadata/ or *Datasets as Topic/ or *Quality Improvement/ or "Information Storage and Retrieval"/st or Data Collection/st

(data ADJ2 (element* or set*) ADJ3 (common or minimum or minimal or requirement*)).ti,ab.

(data ADJ2 (quality or harmon* or standard*)).ti,ab.

(core domain* or best practice* or quality improv*).ti,ab.

**Set#4 – Rare Diseases**

Rare Diseases/ or rare disease/

((orphan or rare or low prevalence) ADJ3 disease*).ti,ab,kf.

Database(s): **Embase**1974 to 2022 September 19**, Ovid MEDLINE(R) ALL**1946 to September 19, 2022
Search Strategy:

| # | Searches | Results |
| --- | --- | --- |
| 1 | ((registry or registries) adj4 (establish* or development or standards* or model* or developing or design or framework* or build* or implement* or quality assurance)).ti. | 1746 |
| 2 | (EPIRARE or Open-Source Registry System for Rare Diseases or (OSSE adj2 framework)).ti,ab. | 27 |
| 3 | 1 or 2 | 1770 |
| 4 | Registries/ or exp patient registry/ | 247855 |
| 5 | (registry or registries).ti,ab,kf. | 438000 |
| 6 | 4 or 5 | 525794 |
| 7 | *Quality Control/ or Data Accuracy/ or Common Data Elements/ or *Metadata/ or *Datasets as Topic/ or *Quality Improvement/ or "Information Storage and Retrieval"/st or Data Collection/st | 30356 |
| 8 | (data adj2 (element* or set*) adj3 (common or minimum or minimal or requirement*)).ti,ab. | 9118 |
| 9 | (data adj2 (quality or harmon* or standard*)).ti,ab. | 94052 |
| 10 | (core domain* or best practice* or quality improv*).ti,ab. | 207660 |
| 11 | or/7-10 | 325039 |
| 12 | Rare Diseases/ or rare disease/ | 58842 |
| 13 | ((orphan or rare or low prevalence) adj3 disease*).ti,ab,kf. | 149426 |
| 14 | 12 or 13 | 184043 |
| 15 | 6 and 11 and 14 | 353 |
| 16 | 3 or 15 | 2097 |
| 17 | limit 16 to yr="2010 -Current" | 1647 |
| 18 | remove duplicates from 17 | 1084 |

**Appendix 2 -Results from the scoping review**

1. Ahern, Susannah, Geoff Sims, Arul Earnest, and Scott C Bell. 2018. "Optimism, opportunities, outcomes: the Australian Cystic Fibrosis Data Registry." *Internal medicine journal* 48 (6): 721-723. <https://dx.doi.org/10.1111/imj.13807>.
2. Ali, Salma Rashid, Jillian Bryce, Yllka Kodra, Domenica Taruscio, Luca Persani, and Syed Faisal Ahmed. 2021. "The Quality Evaluation of Rare Disease Registries-An Assessment of the Essential Features of a Disease Registry." *International journal of environmental research and public health* 18 (22). <https://dx.doi.org/10.3390/ijerph182211968>.
3. Ali, Salma Rashid, Jillian Bryce, Yllka Kodra, Domenica Taruscio, Luca Persani, and Syed Faisal Ahmed. 2021. "The Quality Evaluation of Rare Disease Registries-An Assessment of the Essential Features of a Disease Registry." *International journal of environmental research and public health* 18 (22). <https://dx.doi.org/10.3390/ijerph182211968>.
4. Allen, Alexander, Hannah Patrick, Jorg Ruof, Barbara Buchberger, Leonor Varela-Lema, Janbernd Kirschner, Stefan Braune, Fabian Rosnagel, Emmanuel Gimenez, Xavier Garcia Cusco, and Chantal Guilhaume. 2022. "Development and Pilot Test of the Registry Evaluation and Quality Standards Tool: An Information Technology-Based Tool to Support and Review Registries." *Value In Health : the Journal of the International Society for Pharmacoeconomics and Outcomes Research* 25 (8): 1390-1398. <https://dx.doi.org/10.1016/j.jval.2021.12.018>.
5. Amselem, S., S. Gueguen, J. Weinbach, A. Clement, and P. Landais. 2021. "RaDiCo, the French national research program on rare disease cohorts." *Orphanet Journal of Rare Diseases* 16 (1): 454. <https://dx.doi.org/10.1186/s13023-021-02089-5>
6. Bellgard, M. I., M. W. Sleeman, F. D. Guerrero, S. Fletcher, G. Baynam, J. Goldblatt, Y. Rubinstein, C. Bell, S. Groft, R. Barrero, A. H. Bittles, S. D. Wilton, C. E. Mason, and T. Weeramanthri. 2014. "Rare Disease Research Roadmap: Navigating the bioinformatics and translational challenges for improved patient health outcomes." *Health Policy and Technology* 3 (4): 325-335. <https://dx.doi.org/10.1016/j.hlpt.2014.08.007>.
7. Bellgard, Matthew, Christophe Beroud, Kay Parkinson, Tess Harris, Segolene Ayme, Gareth Baynam, Tarun Weeramanthri, Hugh Dawkins, and Adam Hunter. 2013. "Dispelling myths about rare disease registry system development." *Source code for biology and medicine* 8 (1): 21. <https://dx.doi.org/10.1186/1751-0473-8-21>.
8. Bellgard, Matthew I., Andrew Macgregor, Fred Janon, Adam Harvey, Peter O'Leary, Adam Hunter, and Hugh Dawkins. 2012. "A modular approach to disease registry design: successful adoption of an internet-based rare disease registry." *Human mutation* 33 (10): E2356-66. <https://doi.org/https://dx.doi.org/10.1002/humu.22154>.
9. Bellgard, Matthew I., Kathryn R. Napier, Alan H. Bittles, Jeffrey Szer, Sue Fletcher, Nikolajs Zeps, Adam A. Hunter, and Jack Goldblatt. 2018. "Design of a framework for the deployment of collaborative independent rare disease-centric registries: Gaucher disease registry model." *Blood cells, molecules & diseases* 68: 232-238. <https://dx.doi.org/10.1016/j.bcmd.2017.01.013>.
10. Bellgard, Matthew I., Lee Render, Maciej Radochonski, and Adam Hunter. 2014. "Second generation registry framework." *Source code for biology and medicine* 9: 14. <https://dx.doi.org/10.1186/1751-0473-9-14>.
11. Bellgard, Matthew I., Tom Snelling, and James M. McGree. 2019. "RD-RAP: beyond rare disease patient registries, devising a comprehensive data and analytic framework." *Orphanet journal of rare diseases* 14 (1): 176. <https://dx.doi.org/10.1186/s13023-019-1139-9>.
12. Bettio, Cinzia, Valentina Salsi, Mirko Orsini, Enrico Calanchi, Luca Magnotta, Luca Gagliardelli, June Kinoshita, Sonia Bergamaschi, and Rossella Tupler. 2021. "The Italian National Registry for FSHD: an enhanced data integration and an analytics framework towards Smart Health Care and Precision Medicine for a rare disease." *Orphanet journal of rare diseases* 16 (1): 470. <https://dx.doi.org/10.1186/s13023-021-02100-z>.
13. Biedermann, Patricia, Rose Ong, Alexander Davydov, Alexandra Orlova, Philip Solovyev, Hong Sun, Graham Wetherill, Monika Brand, and Eva-Maria Didden. 2021. "Standardizing registry data to the OMOP Common Data Model: experience from three pulmonary hypertension databases." *BMC medical research methodology* 21 (1): 238. <https://dx.doi.org/10.1186/s12874-021-01434-3>.
14. Blumenthal, Seth. 2019. "The NQRN Registry Maturational Framework: Evaluating the Capability and Use of Clinical Registries." *EGEMS (Washington, DC)* 7 (1): 29. <https://dx.doi.org/10.5334/egems.278>.
15. Boulanger, Vanessa, Marissa Schlemmer, Suzanne Rossov, Allison Seebald, and Pamela Gavin. 2020. "Establishing Patient Registries for Rare Diseases: Rationale and Challenges." *Pharmaceutical medicine* 34 (3): 185-190. <https://dx.doi.org/10.1007/s40290-020-00332-1>.
16. Busner, J., G. Pandina, S. Z. Domingo, A. K. Berger, M. T. Acosta, N. Fisseha, J. Horrigan, J. Ivkovic, W. Jacobson, D. Revicki, and V. Villalta-Gil. 2021. "Clinician-and Patient-reported Endpoints in CNS Orphan Drug Clinical Trials: ISCTM Position Paper on Best Practices for Endpoint Selection, Validation, Training, and Standardization." *Innovations in Clinical Neuroscience* 18 (10-12): 15-22. <https://www.ncbi.nlm.nih.gov/pmc/articles/PMC8794479/pdf/icns_18_10-12_15.pdf>
17. Choquet, Remy, Meriem Maaroufi, Albane de Carrara, Claude Messiaen, Emmanuel Luigi, and Paul Landais. 2015. "A methodology for a minimum data set for rare diseases to support national centers of excellence for healthcare and research." *Journal of the American Medical Informatics Association : JAMIA* 22 (1): 76-85. <https://dx.doi.org/10.1136/amiajnl-2014-002794>.
18. Chorostowska-Wynimko, Joanna, Marion Wencker, and Ildiko Horvath. 2019. "The importance of effective registries in pulmonary diseases and how to optimize their output." *Chronic respiratory disease* 16: 1479973119881777. <https://dx.doi.org/10.1177/1479973119881777>.
19. Coi, Alessio, Michele Santoro, Ana Villaverde-Hueso, Michele Lipucci Di Paola, Sabina Gainotti, Domenica Taruscio, Manuel Posada de la Paz, and Fabrizio Bianchi. 2016. "The Quality of Rare Disease Registries: Evaluation and Characterization." *Public health genomics* 19 (2): 108-15. <https://dx.doi.org/10.1159/000444476>.
20. DAMA U.K. Working Group. 2013. *The Six Primary Dimensions For Data Quality Assessment, Defining Data Quality Dimensions.* <https://www.sbctc.edu/resources/documents/colleges-staff/commissions-councils/dgc/data-quality-deminsions.pdf>.
21. Daneshvari, Shamsi, Sarah Youssof, and Philip J. Kroth. 2013. "The NIH Office of Rare Diseases Research patient registry Standard: a report from the University of New Mexico's Oculopharyngeal Muscular Dystrophy Patient Registry." *AMIA ... Annual Symposium proceedings. AMIA Symposium* 2013: 269-77. PMID: [24551336](https://pubmed.ncbi.nlm.nih.gov/24551336)
22. Derayeh, Simin, Alireza Kazemi, Reza Rabiei, Azamossadat Hosseini, and Hamid Moghaddasi. 2018. "National information system for rare diseases with an approach to data architecture: A systematic review." *Intractable & rare diseases research* 7 (3): 156-163. <https://dx.doi.org/10.5582/irdr.2018.01065>.
23. Deserno, Thomas M., Daniel Haak, Vincent Brandenburg, Verena Deserno, Christoph Classen, and Paula Specht. 2014. "Integrated image data and medical record management for rare disease registries. A general framework and its instantiation to theGerman Calciphylaxis Registry." *Journal of digital imaging* 27 (6): 702-13. <https://dx.doi.org/10.1007/s10278-014-9698-8>.
24. EUCERD. *EUCERD Core Recommendations on Rare Disease Patient Registration and Data Collection* 2013. European Union Committee of Experts on Rare Diseases. <http://www.rd-action.eu/eucerd/EUCERD_Recommendations/EUCERD_Recommendations_RDRegistryDataCollection_adopted.pdf> (accessed September 28, 2023)
25. European Medicines Agency. 2018. *Discussion paper: Use of patient disease registries for regulatory purposes – methodological and operational considerations.* The Cross-Committee Task Force on Patient Registries. [Use of patient disease registries for regulatory purposes - European Union (europa.eu)](https://encepp.europa.eu/newsroom/news/use-patient-disease-registries-regulatory-purposes-2018-11-12_en).
26. European Medicines Agency. 2021. *Guideline on registry‐based studies.* <https://www.ema.europa.eu/en/documents/scientific-guideline/guideline-registry-based-studies_en-0.pdf>.
27. EURORDIS-NORD-CORD. 2012. *EURORDIS-NORD-CORD Joint Declaration of 10 Key Principles for Rare Disease Patient Registries.* <https://download2.eurordis.org/documents/pdf/EURORDIS_NORD_CORD_JointDec_Registries_FINAL.pdf>.
28. U.S. Food and Drug Administration. 2021. *Real-World Data: Assessing Registries to Support Regulatory Decision-Making for Drug and Biological Products Guidance for Industry: Draft Guidance.* (Rockville, MD: Center for Drug Evaluation and Research, Center for Biologics Evaluation and Research and Oncology Center of Excellence). <https://www.regulations.gov/document/FDA-2021-D-1146-0041>
29. Gainotti, Sabina, Paola Torreri, Chiuhui Mary Wang, Robert Reihs, Heimo Mueller, Emma Heslop, Marco Roos, Dorota Mazena Badowska, Federico de Paulis, Yllka Kodra, Claudio Carta, Estrella Lopez Martin, Vanessa Rangel Miller, Mirella Filocamo, Marina Mora, Mark Thompson, Yaffa Rubinstein, Manuel Posada de la Paz, Lucia Monaco, Hanns Lochmuller, and Domenica Taruscio. 2018. "The RD-Connect Registry & Biobank Finder: a tool for sharing aggregated data and metadata among rare disease researchers." *European journal of human genetics : EJHG* 26 (5): 631-643. <https://dx.doi.org/10.1038/s41431-017-0085-z>.
30. Garcia, Monique, Jenny Downs, Alyce Russell, and Wei Wang. 2018. "Impact of biobanks on research outcomes in rare diseases: a systematic review." *Orphanet journal of rare diseases* 13 (1): 202. <https://dx.doi.org/10.1186/s13023-018-0942-z>.
31. Gliklich, R. E., M. B. Leavy, and N. A. Dreyer. 2020. *Registries for evaluating patient outcomes: a user’s guide (4th Eds.) (Prepared by L&M Policy Research, LLC under Contract No. 290-2014-00004-C with partners OM1 and IQVIA)* Agency for Healthcare Research and Quality (Rockville, MD). <https://effectivehealthcare.ahrq.gov/sites/default/files/pdf/registries-evaluating-patient-outcomes-4th-edition.pdf>.
32. Groenen, Karlijn H. J., Annika Jacobsen, Martijn G. Kersloot, Bruna Dos Santos Vieira, Esther van Enckevort, Rajaram Kaliyaperumal, Derk L. Arts, Peter A. C. t Hoen, Ronald Cornet, Marco Roos, and Leo Schultze Kool. 2021. "The de novo FAIRification process of a registry for vascular anomalies." *Orphanet journal of rare diseases* 16 (1): 376. <https://dx.doi.org/10.1186/s13023-021-02004-y>.
33. Hageman, I. C., H. J. J. van der Steeg, E. Jenetzky, M. Trajanovska, S. K. King, I. de Blaauw, and Ialm van Rooij. 2023. "A Quality Assessment of the ARM-Net Registry Design and Data Collection." *Journal of Pediatric Surgery* 25: 25. <https://dx.doi.org/10.1016/j.jpedsurg.2023.02.049>.
34. Hessl, D., H. Rosselot, R. Miller, G. Espinal, J. Famula, S. L. Sherman, P. K. Todd, A. M. C. Herrera, K. Lipworth, J. Cohen, D. A. Hall, M. Leehey, J. Grigsby, J. D. Weber, S. Alusi, A. Wheeler, M. Raspa, T. Hudson, and S. K. Sobrian. 2022. "The International Fragile X Premutation Registry: building a resource for research and clinical trial readiness." *Journal of Medical Genetics* 59(12): 1165-1170. <https://dx.doi.org/10.1136/jmedgenet-2022-108568>.
35. Hooshafza, S., L. Mc Quaid, G. Stephens, R. Flynn, and L. O'Connor. 2022. "Development of a framework to assess the quality of data sources in healthcare settings." *J Am Med Inform Assoc* 29 (5): 944-952. <https://doi.org/10.1093/jamia/ocac017>.
36. Isaacman, D., O. Iliach, J. Keefer, D. M. Campion, and B. Kelly. 2019. *Registries for rare diseases: a foundation for multi-arm, multi-company trials.* <https://www.iqvia.com/library/white-papers/registries-for-rare-diseases>.
37. Jonker, C. J., S. T. de Vries, H. M. van den Berg, P. McGettigan, A. W. Hoes, and P. G. M. Mol. 2021. "Capturing Data in Rare Disease Registries to Support Regulatory Decision Making: A Survey Study Among Industry and Other Stakeholders." *Drug Saf* 44 (8): 853-861. <https://doi.org/10.1007/s40264-021-01081-z>.
38. Kaliyaperumal, Rajaram, Mark D. Wilkinson, Pablo Alarcon Moreno, Nirupama Benis, Ronald Cornet, Bruna Dos Santos Vieira, Michel Dumontier, Cesar Henrique Bernabe, Annika Jacobsen, Clemence M. A. Le Cornec, Mario Prieto Godoy, Nuria Queralt-Rosinach, Leo J. Schultze Kool, Morris A. Swertz, Philip van Damme, K. Joeri van der Velde, Nawel Lalout, Shuxin Zhang, and Marco Roos. 2022. "Semantic modelling of common data elements for rare disease registries, and a prototype workflow for their deployment over registry data." *Journal of biomedical semantics* 13 (1): 9. <https://dx.doi.org/10.1186/s13326-022-00264-6>.
39. Kinsner-Ovaskainen, Agnieszka, Monica Lanzoni, Ester Garne, Maria Loane, Joan Morris, Amanda Neville, Ciaran Nicholl, Judith Rankin, Anke Rissmann, David Tucker, and Simona Martin. 2018. "A sustainable solution for the activities of the European network for surveillance of congenital anomalies: EUROCAT as part of the EU Platform on Rare Diseases Registration." *European journal of medical genetics* 61 (9): 513-517. <https://dx.doi.org/10.1016/j.ejmg.2018.03.008>.
40. Kodra, Yllka, Jerome Weinbach, Manuel Posada-de-la-Paz, Alessio Coi, S. Lydie Lemonnier, David van Enckevort, Marco Roos, Annika Jacobsen, Ronald Cornet, S. Faisal Ahmed, Virginie Bros-Facer, Veronica Popa, Marieke Van Meel, Daniel Renault, Rainald von Gizycki, Michele Santoro, Paul Landais, Paola Torreri, Claudio Carta, Deborah Mascalzoni, Sabina Gainotti, Estrella Lopez, Anna Ambrosini, Heimo Muller, Robert Reis, Fabrizio Bianchi, Yaffa R. Rubinstein, Hanns Lochmuller, and Domenica Taruscio. 2018. "Recommendations for Improving the Quality of Rare Disease Registries." *International journal of environmental research and public health* 15 (8). <https://dx.doi.org/10.3390/ijerph15081644>.
41. Kourime, M., J. Bryce, J. Jiang, R. Nixon, M. Rodie, and S. F. Ahmed. 2017. "An assessment of the quality of the I-DSD and the I-CAH registries - international registries for rare conditions affecting sex development." *Orphanet journal of rare diseases* 12 (1): 56. <https://dx.doi.org/10.1186/s13023-017-0603-7>.
42. Lautenschlager, Ronald, Florian Kohlmayer, Fabian Prasser, and Klaus A. Kuhn. 2015. "A generic solution for web-based management of pseudonymized data." *BMC medical informatics and decision making* 15: 100. <https://dx.doi.org/10.1186/s12911-015-0222-y>.
43. Liu, Peng, Mengchun Gong, Jie Li, Gareth Baynam, Weiguo Zhu, Yicheng Zhu, Limeng Chen, Weihong Gu, and Shuyang Zhang. 2021. "Innovation in Informatics to Improve Clinical Care and Drug Accessibility for Rare Diseases in China." *Frontiers in pharmacology* 12: 719415. <https://dx.doi.org/10.3389/fphar.2021.719415>.
44. Maaroufi, Meriem, Remy Choquet, Paul Landais, and Marie-Christine Jaulent. 2015. "Towards data integration automation for the French rare disease registry." *AMIA ... Annual Symposium proceedings. AMIA Symposium* 2015: 880-5. PMID: [26958224](https://pubmed.ncbi.nlm.nih.gov/26958224)
45. Marques, Joao Pedro, Ana Luisa Carvalho, Jose Henriques, Joaquim Neto Murta, Jorge Saraiva, and Rufino Silva. 2020. "Design, development and deployment of a web-based interoperable registry for inherited retinal dystrophies in Portugal: the IRD-PT." *Orphanet journal of rare diseases* 15 (1): 304. <https://dx.doi.org/10.1186/s13023-020-01591-6>.
46. Marques, Joao Pedro, Sara Vaz-Pereira, Jose Costa, Ana Marta, Jose Henriques, and Rufino Silva. 2022. "Challenges, facilitators and barriers to the adoption and use of a web-based national IRD registry: lessons learned from the IRD-PT registry." *Orphanet journal of rare diseases* 17 (1): 323. <https://dx.doi.org/10.1186/s13023-022-02489-1>.
47. Maruf, N., and G. Chanchu. 2022. *Planning a rare disease registry.* <https://orphan-reach.com/planning-a-rare-disease-registry/>.
48. McGlinn, Kris, Matthew A. Rutherford, Karl Gisslander, Lucy Hederman, Mark A. Little, and Declan O'Sullivan. 2022. "FAIRVASC: A semantic web approach to rare disease registry integration." *Computers in biology and medicine* 145: 105313. <https://dx.doi.org/10.1016/j.compbiomed.2022.105313>.
49. Mordenti, M., M. Boarini, F. D'Alessandro, E. Pedrini, M. Locatelli, and L. Sangiorgi. 2022. "Remodeling an existing rare disease registry to be used in regulatory context: Lessons learned and recommendations." *Frontiers in Pharmacology* 13 (no pagination). <https://dx.doi.org/10.3389/fphar.2022.966081>.
50. Mullin, A. P., D. Corey, E. C. Turner, R. Liwski, D. Olson, J. Burton, S. Sivakumaran, L. D. Hudson, K. Romero, D. T. Stephenson, and J. Larkindale. 2021. "Standardized Data Structures in Rare Diseases: CDISC User Guides for Duchenne Muscular Dystrophy and Huntington's Disease." *Clinical and Translational Science* 14 (1): 214-221. <https://dx.doi.org/10.1111/cts.12845>.
51. National Cancer Registry Ireland. 2007. *Data confidentiality in the National Cancer Registry.* <https://www.ncri.ie/data.cgi/html/confidentialitypolicy.shtml>.
52. Pericleous, M., C. Kelly, M. Schilsky, A. Dhawan, and A. Ala. 2022. "Defining and characterising a toolkit for the development of a successful European registry for rare liver diseases: a model for building a rare disease registry." *Clinical Medicine, Journal of the Royal College of Physicians of London* 22 (4). <https://dx.doi.org/10.7861/CLINMED.2021-0725>.
53. Rare Disease Task Force. 2011. *Patient registries in the field of rare diseases: overview of the issues surrounding the establishment, management, governance and financing of academic registries.* <https://www.orpha.net/actor/EuropaNews/2011/doc/RDTFReportRegistries2009Rev2011.pdf>.
54. Roos, Marco, Estrella Lopez Martin, and Mark D. Wilkinson. 2017. "Preparing Data at the Source to Foster Interoperability across Rare Disease Resources." *Advances in experimental medicine and biology* 1031: 165-179. <https://dx.doi.org/10.1007/978-3-319-67144-4_9>.
55. Rubinstein, Yaffa R., Stephen C. Groft, Ronald Bartek, Kyle Brown, Ronald A. Christensen, Elaine Collier, Amy Farber, Jennifer Farmer, John H. Ferguson, Christopher B. Forrest, Nicole C. Lockhart, Kate R. McCurdy, Helen Moore, Geraldine B. Pollen, Rachel Richesson, Vanessa Rangel Miller, Sara Hull, and Jim Vaught. 2010. "Creating a global rare disease patient registry linked to a rare diseases biorepository database: Rare Disease-HUB (RD-HUB)." *Contemporary clinical trials* 31 (5): 394-404. <https://dx.doi.org/10.1016/j.cct.2010.06.007>.
56. Rubinstein, Yaffa R., and Pamela McInnes. 2015. "NIH/NCATS/GRDR R Common Data Elements: A leading force for standardized data collection." *Contemporary clinical trials* 42: 78-80. <https://dx.doi.org/10.1016/j.cct.2015.03.003>.
57. Santoro, Michele, Alessio Coi, Michele Lipucci Di Paola, Anna Maria Bianucci, Sabina Gainotti, Emanuela Mollo, Domenica Taruscio, Luciano Vittozzi, and Fabrizio Bianchi. 2015. "Rare disease registries classification and characterization: a data mining approach." *Public health genomics* 18 (2): 113-22. <https://dx.doi.org/10.1159/000369993>.
58. Sernadela, Pedro, Lorena Gonzalez-Castro, Claudio Carta, Eelke van der Horst, Pedro Lopes, Rajaram Kaliyaperumal, Mark Thompson, Rachel Thompson, Nuria Queralt-Rosinach, Estrella Lopez, Libby Wood, Agata Robertson, Claudia Lamanna, Mette Gilling, Michael Orth, Roxana Merino-Martinez, Manuel Posada, Domenica Taruscio, Hanns Lochmuller, Peter Robinson, Marco Roos, and Jose Luis Oliveira. 2017. "Linked Registries: Connecting Rare Diseases Patient Registries through a Semantic Web Layer." *BioMed research international* 2017: 8327980. <https://dx.doi.org/10.1155/2017/8327980>.
59. Song, Peipei, Jiangjiang He, Fen Li, and Chunlin Jin. 2017. "Innovative measures to combat rare diseases in China: The national rare diseases registry system, larger-scale clinical cohort studies, and studies in combination with precision medicine research." *Intractable & rare diseases research* 6 (1): 1-5. <https://dx.doi.org/10.5582/irdr.2017.01003>.
60. Stanimirovic, Dalibor, Eva Murko, Tadej Battelino, and Urh Groselj. 2019. "Development of a pilot rare disease registry: a focus group study of initial steps towards the establishment of a rare disease ecosystem in Slovenia." *Orphanet Journal of Rare Diseases* 14 (1): 172. <https://doi.org/10.1186/s13023-019-1146-x>.
61. Taruscio, Domenica, Emanuela Mollo, Sabina Gainotti, Manuel Posada de la Paz, Fabrizio Bianchi, and Luciano Vittozzi. 2014. "The EPIRARE proposal of a set of indicators and common data elements for the European platform for rare disease registration." *Archives of public health = Archives belges de sante publique* 72 (1): 35. <https://doi.org/https://dx.doi.org/10.1186/2049-3258-72-35>.
62. Vasseur, Jessica, Axel Zieschank, Jens Gobel, Jannik Schaaf, Mareike Dahmer-Heath, Jens Konig, Dennis Kadioglu, and Holger Storf. 2022. "Development of an Interactive Dashboard for OSSE Rare Disease Registries." *Studies in health technology and informatics* 293: 187-188. <https://dx.doi.org/10.3233/SHTI220367>.
63. Vitale, A., F. Della Casa, G. Lopalco, R. M. Pereira, P. Ruscitti, R. Giacomelli, G. Ragab, F. La Torre, E. Bartoloni, E. Del Giudice, C. Lomater, G. Emmi, M. Govoni, M. C. Maggio, A. Maier, J. Makowska, B. Ogunjimi, P. P. Sfikakis, P. Sfriso, C. Gaggiano, F. Iannone, M. A. Dagostin, I. Di Cola, L. Navarini, A. A. Ahmed Mahmoud, F. Cardinale, I. Riccucci, M. P. Paroli, E. M. Marucco, I. Mattioli, J. Sota, A. Abbruzzese, I. P. B. Antonelli, P. Cipriani, A. Tufan, C. Fabiani, M. M. Ramadan, M. Cattalini, R. C. Kardas, G. D. Sebastiani, H. A. M. Giardini, J. Hernandez-Rodriguez, V. Mastrorilli, E. Wiesik-Szewczyk, M. Frassi, V. Caggiano, S. Telesca, H. F. Giordano, E. Guadalupi, T. Giani, A. Renieri, S. Colella, G. Cataldi, M. Gentile, A. Fabbiani, I. A. Al-Maghlouth, B. Frediani, A. Balistreri, D. Rigante, and L. Cantarini. 2022. "Development and Implementation of the AIDA International Registry for Patients With Still's Disease." *Frontiers in Medicine* 9: 878797. <https:doi.org/10.3389/fmed.2022.878797>.
64. Zaletel, Metka, and Marcel Kralj. 2015. *Methodological guidelines and recommendations for efficient and rational governance of patient registries.* National Institute of Public Health, Ljubljana. (Trubarjeva 2 National Institute of Public Health, 1000 Ljubljana, Slovenia). <https://health.ec.europa.eu/system/files/2016-11/patient_registries_guidelines_en_0.pdf>
