# Supplementary material for: Scoping review of the recommendations and guidance for improving the quality of rare disease registries
Source: Orphanet J Rare Dis. 2024 May 6;19:187. doi: 10.1186/s13023-024-03193-y (PMC11075280; doi:10.1186/s13023-024-03193-y)
Supplement: Supplementary file 2 — Supplementary Material 2 [file 13023_2024_3193_MOESM2_ESM.docx]

February 22, 2024

Anneliene Hechtelt Jonker

Orphanet Journal of Rare Diseases

**Reference: OJRD-D-23-00913**

Dear Dr. Jonker,

On behalf of my co-authors, I am very pleased to resubmit a revised version of our paper titled: *“Scoping review of best practices for improving the quality of rare disease registries*”. We would like to thank the reviewers for their careful review of our paper, which made it stronger as a result.

Two versions of the revised manuscript have been uploaded (one version using track changes and one clean version with all changes accepted) as well as our point-by-point responses to each comment raised by the two reviewers (also given below).

We look forward to hearing from you.

Sincerely,

Jean-Éric Tarride, PhD

Professor, Department of Health Research Methods, Evidence, and Impact

McMaster Chair for Health Technology Management

Director, Centre for Health Economics and Policy Analysis (CHEPA), McMaster University

Director, Programs for Assessment of Technology in Health (PATH) Research Institute

**RESPONSE TO COMMENTS**

**REVIEWER 1**

Reviewer #1: In this manuscript, the authors conducted a literature review on rare disease registries (RDRs) with the aim of identifying best practices to improve the quality of RDRs. The authors concluded by describing the characteristics of RDRs, including three main domains and several subdomains, which are considered to be the best practices for qualifying RDRs. The structure of the review was well organized.

Minor revisions needed before it could be accepted for publication:

1. Title: The use of the term "best practices" in the title may imply that all RDRs included in the review are the best, which may not be appropriate. Consider revising the title to reflect this.

**RESPONSE: we revised the title by changing “best practices” to “the recommendations and guidance” as shown below. Deletions are indicated by s~~trikethrough~~ while underlined text represent new additions.**

***“ SCOPING REVIEW OF THE RECOMMENDATIONS AND GUIDANCE ~~BEST PRACTICES~~ FOR IMPROVING THE QUALITY OF RARE DISEASE REGISTRIES”***

2. Abstract: In line 43, consider using "determine" instead of "reduce"

**RESPONSE: we now use the term “determine” instead of “reduce” as show below. Deletions are indicated by s~~trikethrough~~ while underlined text represent new additions.**

***“However, they often vary qualitatively, structurally, and operationally in ways that can determine ~~reduce~~ their potential utility as a source of evidence to support decision-making regarding the approval and funding of new treatments for rare diseases.”***

3. The discrepancy in the number of references yielded should be addressed by verifying the numbers and ensuring consistency with the "RESULTS" section and Figure 1.

**RESPONSE:**

- **The total number of references in the manuscript is greater than the number of references identified through the literature search as additional sources were used to develop the introduction and discussion sections.**
- **To rectify this potential concern, we have added an Appendix 2, which lists the literature identified through the literature search. We also added in the results of the search strategy section, the following underlined text.**

***“…Figure 1 presents the PRISMA diagram summarizing the screening process and key reasons for exclusion. Appendix 2 presents the list of the 64 documents identified through the literature review and used to develop the framework.”***

4. Additionally, when using "FAIR" for the first time in line 58, please give the complete words.

**RESPONSE: we have provided in the acronyms of FAIR in the abstract as shown below by the underlined text.**

***“…and 3) information technology (IT) infrastructure (physical and virtual infrastructure; and software infrastructure guided by FAIR principles (Findability; Accessibility; Interoperability; and Reusability).”***

5. The "RESULTS" section is well organized.

**RESPONSE: Thank you for the feedback.**

6. The "Discussion" section should be revised to provide a more in-depth analysis of the advantages and disadvantages of different designs in various guidelines and explore possible improvements for RDRs.

**RESPONSE:**

- **To answer this comment, we have added to the text a discussion on the similarities and differences of our framework compared to Kodra et al. (2018), Ali et al. (2020) and the European Medicine Agency (EMA) (2021). As already mentioned in the discussion section, very few papers provide recommendations on all the elements required to set up and maintain high-quality RDRs. In addition to Kodra et al. (2018) and Ali et al. (2020), the 2021 EMA guidance, which is now described, is the only other document providing an overall guidance.**
- **The following has been added to the discussion to compare our framework to these three guidelines. Deletions are indicated by s~~trikethrough~~ while underlined text represent new additions.**

***While many guidelines focused on certain dimensions of RDRs’ quality (e.g., governance, core data elements), only three papers provided overall guidance on an extensive set of elements required to set up and maintain high-quality RDRs. Among those, in 2018, ~~the Italian National Center of Rare Diseases in collaboration with other European countries published~~ Kodra et al. 2018 reported on the set of 17 recommendations to improve the quality of RDRs[25] developed by a select group of experts convened by the Italian National Center of Rare Diseases in collaboration with other European countries [25]……….. The 2021 European Medicine Agency (EMA) guidance [49] integrated guidelines from PARENT Joint Action Methodological Guidance [21], the EUnetHTA’s Registry Evaluation and Quality Standards Tool (REQueST) [20], the US Agency for Healthcare Research and Quality (AHRQ)’s Users’ Guide on registries [12], and the European Reference Network Patient Registry Platform [91]. The EMA guidance provides information regarding two main domains: Administrative Information (subdomains include governance, consent and data protection) and Methods (subdomains include objectives, data providers, population, data elements, infrastructure, quality requirements). Our review broadly aligns with all three of these sources in terms of content, but it is more closely aligned with Ali et al. 2021 in terms of organization and number of domains (registry governance, data*** ***quality, IT infrastructure). However, compared to these guidances based on consensus panels or surveys, the evidence leading to our framework was based on a scoping review of the literature and we synthesized a broader set of quality indicators deemed essential by the literature.”***

- Since the FDA released an update to its guidance regarding the use of registry data for regulatory decision making in December 2023, we also added the following to the discussion section immediately after the previous section.

***In December 2023, the FDA released its finalized real-world data guidance regarding a registry’s fitness to support regulatory decision making, which is consistent with our framework (e.g., governance, data, information technology infrastructure). However, this guidance was published after the completion of the scoping review and as such was not included in this review.***

**REVIEWER #2**

Reviewer #2: It's a scoping paper about the literature on rare disease registries to improve best practices to improve the quality of rare disease registries based on a Canadian view. It refers to European projects and networks like EUneHTA, the EUnetHTA  Registry Evaluation and Quality Standards Tool (REQueST) and to the EMA guideline on registry-based studies.

1. Unfortunately it does not at all refer to the European Platform on Rare Disease Registration (EU RD Platform, Aim of the Platform | EU RD Platform (europa.eu)) which main objective is to cope with the enormous fragmentation of rare disease (RD) patients data contained in hundreds of registries across Europe - a similar problem addressed by this paper. This EU RD Platform has developed a set of common data elements for rare diseases registration (EU_RD_Platform_CDS_Final.pdf (europa.eu)). This is based on the European RD Registry Infrastructure (ERDRI), which supports existing registries and the creation of new registries. The EU RD Platform sets EU-level standards for RD data collection and data exchange and provides training on the use of the tools and services offered. In addition to ERDRI, the EU RD Platform includes a data repository which consist of the JRC-EUROCAT Central Registry on congenital anomalies in Europe. The EU RD Platform is open to all RD registries. Its final goal is to act as a knowledge generation center benefiting healthcare providers including European Reference Networks, researchers, patients and policy-makers in the common effort to improve diagnosis and treatment for patients living with a rare disease.

**RESPONSE**

- **The objective of our scoping review was to identify standards, guidance, or recommendations for improving the quality of individual existing registries. As such we did not review the evidence related to specific centralized/distributed infrastructure of a group of registries.**
- **Nonetheless, our search strategy did capture the European Platform on Rare Disease Registration and several sources identified through our search strategy were included in the scoping review.**
  a. **Reference 59: Kaliyaperumal R, Wilkinson MD, Moreno PA, Benis N, Cornet R, Dos Santos Vieira B, et al. Semantic modelling of common data elements for rare disease registries, and a prototype workflow for their deployment over registry data. Journal of biomedical semantics. 2022;13(1):9. doi:** [**https://dx.doi.org/10.1186/s13326-022-00264-6**](https://dx.doi.org/10.1186/s13326-022-00264-6)**.**
  b. **Reference 46. Kinsner-Ovaskainen A, Lanzoni M, Garne E, Loane M, Morris J, Neville A, et al. A sustainable solution for the activities of the European network for surveillance of congenital anomalies: EUROCAT as part of the EU Platform on Rare Diseases Registration. Eur J Med Genet. 2018;61(9):513-7. doi: <https://dx.doi.org/10.1016/j.ejmg.2018.03.008>.**
  c. **Reference 79. Taruscio D, Mollo E, Gainotti S, Posada de la Paz M, Bianchi F, Vittozzi L. The EPIRARE proposal of a set of indicators and common data elements for the European platform for rare disease registration. Archives of public health = Archives belges de sante publique. 2014;72(1):35. doi:** [**https://dx.doi.org/10.1186/2049-3258-72-35**](https://dx.doi.org/10.1186/2049-3258-72-35)**.**
- **As part of our screening process, we also identified two additional references referring to the European Platform on Rare Disease Registration, which were ultimately excluded from the scoping review for the following reasons.**
  a. **The paper by Abaza et al. 2022 (Domain-Specific Common Data Elements for Rare Disease Registration: Conceptual Approach of a European Joint Initiative Toward Semantic Interoperability in Rare Disease Research. JMIR medical informatics. 2022;10(5):e32158.) was excluded following a full text review as this paper reported on a process for identifying Domain Common Data Elements (DCDE) to enhance the EU RD platform CDE, which was beyond the scope of the paper. Our focus was not on developing specific CDE or DCDE but rather on identifying those papers that offered specific standards, guidance, or recommendations for improving the quality of individual existing registries. Abaza H, Kadioglu D, Martin S, Papadopoulou A, Dos Santos Vieira B, Schaefer F, et al doi: <https://dx.doi.org/10.2196/32158>.**
  b. **EU_RD_Platform_CDS_Final.pdf was captured through the grey literature search but it was excluded from our scoping review as this was a website providing a list of CDEs without any guidance.**
- **While our paper included sources that referenced the EU RD Platform, we did not specifically discuss the EU RD Platform since the objective of this paper was not to suggest the adoption of or need of a specific centralized infrastructure such as the EU RD platform, or to review systems or processes for multi-registry platforms. Rather, the objective of our study was to identify specific standards, guidance, or recommendations to support quality improvement of individual existing registries.**
- **However, to address the reviewer’s comment we added the following at the end of the discussion section and cited the EU RD Platform and the Abaza paper for their discussion of the platform:**

***As a next step, for RDRs interested in the harmonization of their data collection with other registries, the European Platform on Rare Disease Registration (EU RD Platform) serves as an example of how this might be achieved. With over 600 diverse and fragmented rare disease registries in Europe, the European Commission’s Joint Research Centre in collaboration with stakeholders took on the tremendous task of establishing standards for integration, training, and interoperability of RDR data across Europe. A core element of the EU RD Platform is the European Rare Disease Registry Infrastructure (ERDRI), which consists of a directory of registries, a data repository, a pseudonymization tool and importantly the EU RD Platform, comprising a set of 16 common data elements that capture the characteristics of rare disease patients such as demographic, clinical, diagnostic, and genetic information.***

2. Therefore it would be good to assess/compare the criteria with the different subdomains of the REQueST tool of this Canadian paper with the set of recommendations for rare disease registration from the EU RD Platform. Only some older European guidelines (i.e. from 2018) are mentioned.

**RESPONSE**

- **Although we agree that an assessment of the EU RD Platform with the subdomains of the REQueST tool may be of value, it is beyond the scope of this paper.**
- **However, as per a comment from another reviewer, we are comparing our framework with three other documents included in our scoping review that provided recommendations on all the elements required to set up and maintain high-quality RDRs frameworks: Kodra et al. (2018); Ali et al. (2020); and the EMA (2021).**
- **The following has been added to the discussion to compare our framework to these three guidelines. Deletions are indicated by s~~trikethrough~~ while underlined text represent new additions.**

***While many guidelines focused on certain dimensions of RDRs’ quality (e.g., governance, core data elements), only three papers provided overall guidance on all the elements required to set up and maintain high-quality RDRs. Among those, in 2018, ~~the Italian National Center of Rare Diseases in collaboration with other European countries published~~ Kodra et al. 2018 reported on the set of 17 recommendations to improve the quality of RDRs[25] developed by a select group of experts convened by the Italian National Center of Rare Diseases in collaboration with other European countries [25]………. The 2021 European Medicine Agency (EMA) guidance [49] integrated guidelines from PARENT Joint Action Methodological Guidance [21], the EUnetHTA’s Registry Evaluation and Quality Standards Tool (REQueST) [20], the US Agency for Healthcare Research and Quality (AHRQ)’s Users’ Guide on registries [12], and the European Reference Network Patient Registry Platform [91]. The EMA guidance provides information regarding two main domains: Administrative Information (subdomains include Governance, Consent and Data Protection) and Methods (subdomains include objectives, data providers, population, data elements, infrastructure, quality requirements). Our paper broadly aligns with all three of these sources in terms of content, but it is more closely aligned with Ali et al. 2021 in terms of organization and number of domains (Registry Governance, Data Quality, IT Infrastructure). However, compared to these guidances based on consensus panels or surveys, the evidence leading to our framework was based on a scoping review of the literature and we synthesized a broader set of quality indicators deemed essential by the literature.***

3. It also would be important to analyze why the search strategy has missed this information of EU RD Platform, ERDRI and the repository.

**RESPONSE:**

- **As per our RESPONSE to comment #1, our search strategy identified several publications related to the EU RD platform and we added a paragraph in our discussion section to refer to the EU RD Platform. Given the reviewer’s concerns that our search strategy may have missed some critical sources, we conducted another quick search on PubMed as well as the website of the EU Science Hub and did not find any additional sources other than the ones we identified.**
- **No other changes were made to the paper.**

4. Further, it would be good to include a paragraph on the Canadian system for Rare disease registries (i.e. funding, purpose etc.)  and to know their current work on rare disease registries. In line 504, 25 Canadian Registries are mentioned. It would be good to have some more information about them. As a conclusion it would be important to have an overview/outline how the Canadian guidelines developed by consensus differ from the European ones and Canada might profit on a cooperation with the European Platform on Rare disease registration. An alignment of processes might be useful.

**RESPONSE**

- **Although we indicated in the introduction that the REQueST tool was used by the Canadian Agency for Drugs and Technologies in Health (CADTH) to describe 25 RDRs based on publicly available information reported by the RDRs[21], only a summary poster is available on the CADTH** website [https://www.cadth.ca/sites/default/files/pdf/es0369-cadth-poster-laurie-lambert-final.pdf] **and there are no public repository of all Canadian RDR registries and their characteristics (e.g. funding, purpose, quality).**
- **To address this comment, we added in the discussion section related to future research the following underlined text.**

***“This review has also identified a few areas which merit further consideration. First, from a Canadian standpoint, future work is needed to develop a database of Canadian RDRs along with information on their key characteristics (e.g., purpose, population, funding) and information regarding governance, data and IT infrastructure. Second,…”***

- **As indicated in our RESPONSE to comment #2, we added in the discussion a comparison of our framework with three other frameworks touching in all components of a RDR registry (including the EMA 2021 recommendations).**
- **Regarding the last comment, it would have not been appropriate for us to advise Canada on any alignment or cooperation with the European Platform on Rare disease registration due to our study objectives, which were “to identify best practices to improve the quality of RDRs”. As such, a review or analysis of a platform such as the European Platform on Rare disease registration was outside of the scope of our work.**
- **No additional changes were made to the paper other than adding in the discussion a paragraph on the European Platform on Rare Disease Registration (EU RD Platform) and comparing our framework with three other frameworks including the EMA framework.**
